# Artificial Intelligence in Radiology—Ethical Considerations

**DOI:** 10.3390/diagnostics10040231

**Published:** 2020-04-17

**Authors:** Adrian P. Brady, Emanuele Neri

**Affiliations:** 1 Radiology Department, Mercy University Hospital, T12 WE28 Cork, Ireland; 2 European Society of Radiology (ESR), Am Gestade 1, 1010 Vienna, Austria; 3 Diagnostic and Interventional Radiology, Department of Translational Research, University of Pisa, Via Roma, 67, 56126 Pisa, Italy; emanuele.neri@med.unipi.it

**Keywords:** artificial intelligence, radiology ethics, machine learning

## Abstract

Artificial intelligence (AI) is poised to change much about the way we practice radiology in the near future. The power of AI tools has the potential to offer substantial benefit to patients. Conversely, there are dangers inherent in the deployment of AI in radiology, if this is done without regard to possible ethical risks. Some ethical issues are obvious; others are less easily discerned, and less easily avoided. This paper explains some of the ethical difficulties of which we are presently aware, and some of the measures we may take to protect against misuse of AI.

## 1. Introduction

The advent of artificial intelligence (AI) applications is likely to be one of the most far-reaching developments in the history of medicine, with implications for all medical specialties, and potentially for all users of healthcare services in the future. As a data-driven specialty, radiology will be at the forefront in terms of integration of AI tools into practice; as a corollary, radiologists may be among those medical practitioners whose way of working will be most impacted by AI. Many media comments and articles have glibly suggested that radiologists are likely to be replaced by AI algorithms in the immediate future [1], and at least one prominent researcher in the field of deep learning has suggested that we should stop training radiologists for the future, as we will soon be superseded in performance by AI [2]. “The role of radiologists will evolve from doing perceptual things that could probably be done by a highly trained pigeon to doing far more cognitive things” was his subsequent clarification of this statement [3], which probably comes closer to describing how AI will change our work. It seems likely that AI will replace many of the time- and labour-intensive aspects of our work (e.g., scrutinizing hundreds of lung window setting images from a chest CT for nodules), freeing us to devote our time and energy to higher-level tasks (e.g., determining the significance of those nodules).

However AI impacts upon patients and radiologists, everybody involved has a responsibility to ensure that the impact is for good, not ill. This may seem self-evident, and barely worth mentioning, but the complexity of AI can result in unexpected issues of ethical concern about how AI algorithms are developed, and how they are used. In recent years, many professional bodies, representing a wide variety of disciplines involved in deep learning and AI applications, have produced ethical statements and suggested codes of ethical behaviour relating to AI; an example of broadly-applicable principles is the set of 23 statements derived from the 2017 Asilomar conference of the Future of Life Institute [4]. With respect to AI in radiology, in 2019 a team of experts representing the European Society of Radiology (ESR), the American College of Radiology (ACR), the Radiological Society of North America (RSNA), the Canadian Association of Radiologists (CAR), the Society for Imaging Informatics in Medicine (SIIM), the European Society of Medical Imaging Informatics (EuSoMII), and the American Association of Physicists in Medicine (AAPM) together wrote a multi-society statement of the ethics of AI in radiology, published as a summary in four leading journals (Insights into Imaging [5], JACR, Radiology and CARJ), with the full statement published on the societies’ websites [6]. The statement considered ethical issues that may arise from many aspects of deep learning, with specific reference to radiology applications. It sought to identify key issues, and to provide guidance on how potential problems can be avoided or solved. Within the full statement, the possible ethical dilemmas or abuses that could arise, deliberately or inadvertently, from AI applications in radiology have been reviewed.

Other national societies have published consensus recommendations on the ethical use of AI, such as the Italian Society of Medical and Interventional Radiology [7], the Canadian Association of Radiologists [8], and the French Radiology Community [9].

The aim of the review is to discuss some technological drawbacks of AI, certain related ethical issues, and to address potential solutions.

## 2. Data Ethical Issues

During the 19th century, vast fortunes were made by those who were able to access and control the supply of steel. During the 20th century, oil was the commodity of choice for monopolization and wealth creation. To date in the 21st century, data represent the route to vast riches. In September 2019, 7 of the largest 8 companies in the world by market capitalization were predominantly active in the area of data (Microsoft, Apple, Amazon, Google, Facebook, Alibaba & Tencent) [10]. Medical data represent fertile ground for profit if they can be accessed and harnessed. In October 2019, Google bid $2.1 billion for Fitbit. It is estimated that 28 petabytes of data are now generated daily by wearable devices. It’s clearly worth a lot of money to somebody.

Huge benefits may flow to humanity from a proper use of medical data, and AI has the potential to usher in an era of personalized medicine, with diagnosis, treatment, and prognostication aided by the ability of algorithms to identify and analyse information previously unknown or unrecognized. But a misuse of data, or of outputs flowing from widespread application of AI tools also has the potential to cause harm.

## 3. Data Ownership and Privacy

AI-based algorithms for use in radiology require access to large volumes of patient data for the purposes of training, testing, and validating the algorithms. Ideally, this data should be annotated (labelled), and have an assigned “ground truth” (i.e., the answer the algorithm should be expected to arrive at if functioning as expected). This annotation and assignment of ground truth can be a very labour-intensive task [11], but first, the data must be available. In effect, the data are usually patient imaging studies. Ethical issues arise in terms of ownership of data, how data are used, and how the privacy of those from whom the data is derived is protected. Under the EU General Data Protection Regulation (GDPR), patients own and control their own sensitive and/or identifiable data, and must give explicit consent for data re-use or sharing [12]. In other parts of the world, individual rights may be superseded by the collective societal welfare, or ownership of imaging data may reside with the entity performing the imaging [6], making data access easier. Under GDPR rules, patients must give their consent if their imaging studies are to be used to train an AI algorithm, and that consent may need to take a dynamic form, being varied or re-obtained for each version of the algorithm [6]. These legal restrictions can inhibit AI research in some parts of the world relative to others, skewing the academic and commercial playing field.

If a company uses private patient data to market a profitable AI product, there is no clear understanding of who should benefit from the profits. It is arguable that the patients whose data are used to develop the tool should share in the bounty, but mechanisms or rules for this do not exist at present. Blockchain technology may assist with apportioning financial gains in the future. As a general principle, it should be accepted that a patient can be considered to have given their consent for others to use their data only if they have been informed of the value (in monetary terms) of those data [6].

Few patients would be content to imagine that their imaging data would be accessible in an identifiable manner to third parties, and therefore full anonymization of data used in AI development is important. However, real anonymization is more complicated than most people imagine. It has been written that “Entities facile with manipulating massive data can likely re-identify just about any radiology exam” [13]. Even fully anonymized datasets may be manipulatable in a manner that would allow their source to be identified; application of facial recognition software to 3D reconstructions of head and neck imaging can restore identification [14]. Various software solutions to these problems have been tried, including “defacing” head and neck datasets [4].

Furthermore, given that large data companies now frequently control both social media platforms and medical AI applications, it is feasible for such an entity to gather data from smartphone use, internet searches, social media use etc., and to match this information with healthcare data, allowing patient identification. This could lead to unwanted advertising, adverse decisions about insurance or other financial matters, or even to extortion of patients who do not want their medical history made public [6].

Ethical use of patient data demands that AI developers and users be aware of these risks, and take all steps possible to protect patient privacy during development and ongoing use of AI tools.

## 4. Data Bias

In March 2020, a Dutch court ruled that a governmental system (SyRI) designed to detect fraud relating to social services, using algorithms based on profiles of people previously caught committing fraud, was in breach of domestic privacy laws and contrary to the European Convention on Human Rights [15]. Part of the problem was that the algorithm applied biases of those who wrote the program, unsupported by hard evidence, leading to innocent individuals being treated as suspects. Similar problems have arisen in the recent past in other countries, notably in AI tools developed in the US to identify criminals likely to re-offend, whose sentencing was influenced by the algorithm (leading to biased treatment of certain ethnic groups).

Data bias may not be deliberate; in most instances, it is likely that algorithm developers are unaware of biases inadvertently introduced into their work. For example, data used to train algorithms may not accurately represent the population on which the algorithm may be used because of the likelihood that the available data used for training over-represent positive findings (clinical data may be more available for positive research findings; data from negative studies are often under-reported) [16]. This can lead to over-fitting, where AI tools over-interpret the incidence of disease. Selection bias can occur if training data are derived from a population that does not accurately represent the population as a whole; this is likely if those data are derived only from a patient cohort attending a specialist centre, and can lead to bias against groups based on age, sex, ethnic origin, height or many other characteristics, with under-reporting or over-reporting of disease in patients on whom AI is used [9].

Dataset shift is a specific subset of selection bias, arising from the fact that data used to train algorithms do not necessarily match the conditions under which future imaging studies will be performed. Radiologists are used to adapting to changing MR field strength, CT slice thickness, breathing motion artefact etc., but these variations may impact greatly on the performance of AI, unless specifically allowed for in training.

Other sources of data bias include curation bias (where humans can choose from which angle to take images, as in ultrasound) and negative set bias (where datasets over-represent positive or interesting examinations, skewing the fact that most radiology examinations are normal) [6].

## 5. Ground Truth

Supervised machine learning, in which an algorithm learns how best to achieve a pre-labelled ground truth outcome, underpins current radiology AI models [6]. However, there are difficulties with the definition of ground truth in some circumstances, which can introduce bias. Radiological examination is often not the final defining part of diagnosis; ultimate diagnosis, and thus ground truth, may depend on other pieces of information, or other investigations. Thus, to label an imaging study as indicating a specific diagnosis may over-call the ability of imaging to diagnose some conditions. Not all ground glass opacification on chest CT is caused by coronavirus infection; other investigations are needed to confirm the diagnosis. If an AI model associates the diagnosis “COVID-19” with ground glass changes, because that is the ground truth diagnosis most commonly assigned to this finding at the time of training of the algorithm, it may over-diagnose coronavirus infection in many other conditions, with management and ethical implications.

Radiological ground truth is commonly a continuum; human radiologists and referrers are often comfortable with soft classifications such as “probably normal”, and with strategies (time, further investigation) to allow more certainty. It is difficult to program such ill-defined concepts into an AI algorithm; mathematics likes certainty and absolutes, and may need artificial lumping of findings into “definitely normal” or “definitely abnormal” categories, thereby reducing the power and accuracy of the AI tool. Furthermore, not all radiologists will come to the same conclusion about an imaging study; ground truth labelling accuracy can be enhanced by combining interpretations by more than one radiologist [17].

## 6. Black Boxes, Transparency, Interpretability and Explainability

Key to the success of AI in medical applications, including in radiology, will be patient trust that the tool is being used safely, and for their benefit. Intrinsically, humans feel the need to understand how decisions that affect them are made. Asking us to devolve decision making to machines or software which operate beyond our understanding may be more than we are willing to accept. The individual wishes of patients with respect to the use of AI tools in their healthcare must be paramount, yet these wishes may not necessarily conform to the logic underpinning computer algorithms. In 2016, Bonnefron et al. reported that participants in studies about decision-making in autonomous vehicles (AVs) approved of utilitarian AVs (which would sacrifice their passengers for the greater good, if faced with a choice of running over pedestrians or sacrificing their occupants), and would like others to buy them, but would themselves prefer to travel in AVs that protected their passengers at all costs [18]. Similar ambivalence in public attitudes towards AI in healthcare is possible. Will the public accept imperfections in AI-driven healthcare as it relates to individuals, in favour of a potential greater good for the population at large? If, for example, medical imaging is purely protocol-driven, and algorithm-interpreted, will there still be room for the practice of common sense, and for balancing individual- and population-based risks relating to radiation exposure against specific patient expectations? If AI-driven radiology is acknowledged to be imperfect (and always in evolution though continuing deep learning), will the public accept it because it is less-costly or less labour-intensive than human-provided radiology? After all, at least in more litigious societies, anything less than perfection in human delivery of medical care seems decreasingly tolerable to the public.

The “black box” element of artificial neural network function introduces a large element of opacity into AI-based decision making [19]. If the practice of medicine becomes one where data (imaging or other) are analysed by a neural network which then dictates the actions to be followed, the doctor becomes no more than an agent of the software, with opposable thumbs; Think for a moment about your reaction if your doctor said, “*I don’t know why you are ill, but my computer says, ‘Take these pills’* or *I don’t know why you are ill, but my computer recommends surgery*” [20]. Expecting patients and doctors to trust the outcomes of such black-box processing without being able to unpick the process or to understand how outcomes (good or bad) are arrived at is not far removed from the notion of abandoning the advances based on evidence-based medicine. This cannot, and should not happen. Therefore, we must ensure that artificial neural network data processing remains accessible to interrogation after the fact (transparency); this presents a challenge for data scientists, and to some extent to the whole process of AI development, but is vital to maintain public and professional trust. One (somewhat extreme) solution would be to only use algorithmic approaches to AI (i.e., excluding the use of self-learning neural networks) in medicine. A 2017 report by the AI Now Institute at New York University recommended that “Core public agencies, such as those responsible for criminal justice, healthcare, welfare and education (e.g., ‘high stakes’ domains) should no longer use ‘black box’ AI and algorithmic systems at a minimum should be available for public auditing, testing, and review, and subject to accountability standards” [21].

Interpretability is the ability to understand the workings of an AI model, explainability the ability to explain in understandable terms what happens when the AI model makes a decision [6]. Both are desirable attributes, but only up to a point. The more transparent and interpretable an algorithm is, the less safe it is from malicious attack or from appropriation of intellectual property. Furthermore, the more explainable an AI-mediated decision is, the less it can be based on true deep learning, harnessing the power of AI. After all, much of the advantage of AI depends on its capacity to perform analyses faster than humans, and to identify relationships that we cannot.

## 7. AI in Practice—Ethical Issues

### 7.1. Resource Inequality

AI is very resource intensive, requiring large amounts of data, skills, and computing power for development and deployment. These resources will not be evenly distributed, leading to the potential that some countries, regions, or subgroups of society may be denied access to the potential benefits arising from AI [6]. The fair use of or access to AI tools is not intrinsic to AI algorithms, and must be carefully monitored. Those individuals and companies engaged in AI development must be financially incentivised to do their work; at the same time, healthcare systems deploying AI tools should strive to ensure that the benefits are delivered to all patients they serve, not just to those with access to greater resources [22]. In the radiological domain the availability of AI solutions should be ensured across countries, despite differences in healthcare systems and availability of radiological equipment.

### 7.2. Liability

At present, if the appropriate standard of care is not provided by a doctor, that doctor can be held legally liable for bad outcomes for patients. Physicians are used to shouldering that responsibility, and in many countries, carry insurance to indemnify themselves against legal judgements that may derive from such events. Matters become more complex when decision-making is guided by, or even entirely devolved to AI. Who is liable for a bad outcome in the AI future? Is it the doctor who used the AI tool, the software developer who designed it, or the hospital which purchased it? Under the recent EU Medical Devices Regulation, “software in its own right, when specifically intended by the manufacturer to be used for one or more of the medical purposes set out in the definition of a medical device, qualifies as a medical device”, and is thereby covered under product liability legislation [23,24]. Conversely, if a radiologist fails to use or abide by the advice of an AI tool in the future, and a poor patient outcome results, it is conceivable that human over-riding of AI may be considered negligence by a court [22].

### 7.3. Conflicts of Interest

Radiologists are, and should be, involved in many commercial companies developing radiology AI tools. Our expertise should be central to determining the use cases on which AI resources should be utilised, and to developing the algorithms and their clinical applications. However, the potential exists for radiologists and others involved in AI development to find themselves in conflict-of-interest situations if and when commercial decisions are being made regarding purchasing and use of AI tools. This is no different from potential conflicts arising from drug or device development, and can be managed by honest use of public disclosure, institutional oversight, divestment of beneficial ownership, or recusal from commercial decisions, as appropriate [6,24,25].

### 7.4. Workforce Disruption

One of the potential societal hazards of AI (which receives much publicity in popular media reporting) is the danger of mass unemployment arising from AI making many traditional jobs redundant [19]. As physicians, we are not immune to this possibility, and the fear arising from it. This could lead to behaviours and practices in the future designed to ensure the continuing relevance and roles of human practitioners in healthcare, regardless of whether or not continued direct human involvement is of ultimate benefit to the public. Much of the current debate about ethical issues surrounding AI usage in healthcare centres around the presumption that one of the key roles of humans in implementation of AI usage is to prevent negative consequences of this implementation. It would be perverse to ignore the possibility that humans may not act disinterestedly, and that we, as radiologists, have a vested interest in ensuring we are not made entirely redundant by emerging technology and artificial intelligence. Furthermore, in a potential future where our position in the hierarchy is threatened or diminished (in favour of information scientists or other non-traditional medical players), we may feel driven to protect what we perceive as our relevance. Not only is there an ethical imperative to protect patients and the general public from the dangers of “robot-only radiology”, there is also a countervailing need for protection against radiologist (or other physician) self-interest, if it conflicts with the general good.

The final words on the danger of workforce disruption can be left to Curt Langlotz: “‘Will AI replace radiologists?’ is the wrong question. The right answer is: Radiologists who use AI will replace radiologists who don’t” [26].

## 8. Solutions: The Algor-Ethics

It is difficult to find immediate solutions to the drawbacks of AI. At this stage one can only propose potential solutions, behaviours that can drive ethical use of AI, as summarised in Table 1. With this purpose in mind, the European Commission established in 2018 the High-level Artificial Intelligence expert group with the general objective of supporting the implementation of the European Strategy on Artificial Intelligence, including the elaboration of recommendations on future-related policy development and on ethical, legal, and societal issues related to AI, including socio-economic challenges. This group recommended that the development, deployment, and use of AI systems should adhere to the ethical principles of *respect for human autonomy, prevention of harm, fairness/equity and explicability* [27]. It is clear that an intrinsically ethical AI could be a solution to avoid some drawbacks, i.e., the case of the Seldon algorithm which places limits to the probability that an algorithm will produce any specified undesirable behaviour [28].

However with the aim of developing a “human- and environment-centered” (instead of a “technologically-centered”) AI, on February 28th 2020, the “Rome call for ethics” charter, promoted by the Pontifical Academy for Life, an advisory body to Pope Francis, was signed by the International Business Machines Corporation (IBM), Microsoft Corporation, and the Food and Agriculture Organization (FAO). [29].

An important sentence of the charter reads as follows “Now more than ever, we must guarantee an outlook in which AI is developed with a focus not on technology, but rather for the good of humanity and of the environment, of our common and shared home and of its human inhabitants, who are inextricably connected. In other words, a vision in which human beings and nature are at the heart of how digital innovation is developed, supported rather than gradually replaced by technologies that behave like rational actors but are in no way human.”

According to the document, “the sponsors of the call express their desire to work together, in this context and at a national and international level, to promote ‘algor-ethics.’” “Algor-ethics,” according to the text, is “the ethical use of artificial intelligence according to the principles of transparency, inclusion, responsibility, impartiality, reliability, security, and privacy”.

## 9. Conclusions

A danger of a paper such as this, highlighting potential ethical difficulties arising from the introduction of new technology, is that it may be too negative, focusing only on what can go wrong. Certainly, widespread use of AI in radiology could lead to unwelcome and negative consequences. However, this in only likely if we are unaware of, or ignore, these possibilities. The main challenge is to anticipate how rapidly-evolving systems may go wrong or could be abused, and to protect against these possible outcomes (ideally before they happen) [30]. Once we can conceive of potential harms, we can protect against them, and strive towards the undoubted benefits achievable from AI. It is with this in mind that the radiology community has devoted considerable attention to developing ethical codes for AI use [6]. As we learn how to use AI, we must ensure that we also learn how to implement it ethically, to realise its potential for the benefit of humanity.

## Figures and Tables

**Table 1:** Ethical issues, technological drawbacks of AI and potential solutions.

Technological Drawbacks of AI	Ethical Implications	Potential Solutions
Black box element of AI	Risk of validating the unknown: i.e., in the radiology report	AI must be accessible to interrogation (transparency) Only use of algorithmic approaches to AI (i.e., excluding the use of self-learning neural networks)
Complexity of AI models	AI models not accessible and not understandable for the radiologist	Need for explicability and interpretability of AI Radiologists should be trained to understand the basic principles of AI and the radiological AI models
AI errors: risk of overfitting*	Impact on radiological diagnosis	Appropriate training of AI: large multicentric trials with clinical validation of the imaging data used for the ground truth
Difficult access to large amount of data for AI training	Ownership of data, how data are used, and how the privacy of those from whom the data is derived is protected	Patients must give their consent if their imaging studies are to be used to train an AI algorithm. Consent should take a dynamic form, being varied or re-obtained for each version of the algorithm Take all steps possible to protect patient privacy during development and ongoing use of AI tools Use of blockchain technology
Automation bias	Radiologist decision may be biased by the AI automation Radiologists tasks may be replaced by AI with consequent workforce disruption	Radiologists should be trained in the appropriate use of AI in clinical practice Radiologists who use AI will replace radiologists who do not

*AI tools over-interpret the incidence of disease, since the data used to train algorithms may not accurately represent the population on which the algorithm may be used.
